# Distribution of bla ^OXA-23^, IS_*Aba*_*, Aminoglycosides* resistant genes among burned & ICU patients in Tehran and Sari, Iran

**DOI:** 10.1186/s12941-014-0038-0

**Published:** 2014-09-25

**Authors:** Mohtaram Nasrolahei, Bahador Zahedi, Abbas Bahador, Hossein Saghi, Soudeh Kholdi, Neda Jalalvand, Davoud Esmaeili

**Affiliations:** Department of Microbiology, Faculty of Medicine, Mazandaran University of Medical Sciences, Sari, Iran; Departments of Microbiology, Faculty of Medicine, Tehran University of Medical Sciences, Tehran, Iran; Applied Microbiology Research center and Microbiology Department, Baqiyatallah University Medical of Sciences, Tehran, Iran; Department of Genetic‚ Science and Research Branch, Islamic Azad University, Tabriz, East Azerbaijan Iran

**Keywords:** *Acinetobacter baumannii*‚ bla ^OXA-23^ ‚ IS_*Aba*_, Carbapenemase, Aminoglycoside

## Abstract

**Background:**

Multidrug resistant strains of *Acinetobacter baumannii* (MDR-AB) have emerged as alarming nosocomial pathogens among patients admitted to Intensive Care Unit and burned patients. The aim of this study was to determine the susceptibility of *A. baumannii* isolates, the carbapenems resistance patterns bla ^OXA-23^ and also IS_*Aba*_ elements of *A. baumannii* isolates among burned and ICU patients in Tehran and Sari, Iran.

**Methods:**

In this study, 100 *A. baumannii* isolates from burned and ICU patients in Tehran and Sari (Iran) during 2013 were tested for determination of antimicrobials susceptibility by the disc-diffusion method on Mueller Hinton agar recommended by the guidelines of Clinical and Laboratory Standards Institute (CLSI), and frequency bla^OXA-23^ carbapenemase genes, and insertion elements IS_*Aba*_ genes were studied by PCR method.

**Results:**

The highest rates of susceptibility were observed with Colistin (88.7%), Tigecycline (82.2%), Imipenem (67%) and IS_Aba_ (32.2%). The extensively drug-resistance and pan drug-resistance were observed in 37.1% and 8.1% isolates, respectively.

Results indicated among isolates resistant to Aminoglycoside and Carbapenem, the highest resistance was observed to Streptomycin (90%) ‚ and the most sensitivity was to Imipenem (67%).

**Conclusions:**

This is the most study that attempted to detect *Acinetobacter baumanii* the insertion elements IS_*Aba*_, bla ^OXA-23^ and aminoglycosides resistance in MDR-AB isolates from burned and ICU patients in Iran. In a timely manner, antimicrobial resistance surveillance and strict infection control strategies are still lacking in burn ward and ICU in Iran, despite the alarming emergence of MDR-AB strains, particularly among those isolates that are not susceptible to Colistin. The results of this study are consistent with a recent report in which a number of combinations exhibited potent activity against Multidrug resistant strains of *A. baumannii* (MDR-AB).

## Background

Multidrug drug-resistant (MDR) and extensively drug-resistance (XDR) strains of *Acinetobacter baumannii* have emerged as formidable nosocomial pathogens among burned patient [1,2].

In developing countries, such as Iran, clinicians face serious challenges in management of burned patients with MDR- *A. baumannii* (MDR-AB) infections, which present significant health care challenges by prolonging hospitalization, treatment failures, and increased mortality [3].

Members of the genus *Acinetobacter* have been implicated in a wide spectrum of infectious diseases such as Respiratory Infection, Bacteremia, Meningitis and Urinary Tract Infection [4]. *Acinetobacter spp* infections in admitted intensive care unit (ICU) patients hospitals very dangerous and acquired resistance of *A. baumannii* to carbapenems has increasingly been reported all over the world during the last decade [5].

The clinical strains of *A. baumannii* are usually multidrug resistant to aminoglycosides, fluoroquinolones, ureidopenicillins and third generation cephalosporins. Carbapenems were considered to be the mostactive antimicrobials against *A. baumannii*. However, Carbapenem resistance is rising and is often associated with a multidrug resistance phenotype [6,7].

Carbapenems are currently the drug of choice; however, clonal outbreaks of carbapenems resistant *A. baumannii* (CR-AB) have led to an inadequacy of therapeutic choices in treating MDR-AB infections among patients in developing countries [8]. While expression of *bla*^OXA^ genes play a major role in carbapenem resistance among CR-AB [9]), *bla*^OXA^ mediated carbapenem resistance requires enhancement of gene expression either by insertion element IS_*Aba*_ [10-12].

Despite a few reports on the distribution and/or frequency of resistance genes among CR-AB isolate from burned patients in Iran [13,14], the scarcity of molecular epidemiologic data has rendered national efforts to control the spread of CR-AB infections unsuccessful. To date, no extensive studies have addressed the distribution of bla ^oxa-23^, IS_*Aba*_ and distribution in MDR-AB strains that cause infection in burned and ICU patients in Iran.

We in this research surveyed susceptibility of *A. baumannii* isolates, the carbapenems and Aminoglycosides resistance patterns, and its association with IS_*Aba*_ elements of *A. baumannii* isolates among burned and ICU patients in Tehran and Sari, Iran. We obtain consent from the patient for the publication of this report.

## Methods

### Bacterial isolates

During 2013, a total of 100 non-repetitive clinical isolates of *A. baumannii* were collected from the burned patients of a tertiary burn and ICU center in Tehran and Sari, Iran as described previously [1]. All of the patients have given his/her consent for the report to be published. All study-related materials before and during the trial were operated in accordance with national and/or local regulations, as well as with ICH good clinical practices (GCPs) guidelines.

### Identification of *Acinetobacter* species

Initially, isolates were identified as *A. baumannii* using API-20NE system [15,16]. All the clinical Isolates were kept at -20°C in CRYOBANK™ (Copan Diagnostics Inc., Canada) until further testing.

### Bacterial identification

During a One year period, 100 specimens *A. baumannii* (MDR) were isolated from patients that were admitted to Intensive Care Unit and burned patients with proved nosocomial infections in Tehran and Sari‚ (Iran). The strains were isolated from the trachea and burned skin. We considered a strain as MDR if it was resistant to two or more antibiotic classes. Patients had no bacterial infections at the time of admitted to hospital. All the samples were confirmed as *A. baumanni* by biochemical Tests.

### Antimicrobial susceptibility tests

Susceptibility to various antimicrobial agents was determined by the disc-diffusion method on Mueller Hinton agar recommended by the guidelines of Clinical and Laboratory Standards Institute (CLSI). Briefly, 0.1 ml of a suspension of the test microorganism (1.5 × 10 ^8^ cfu /ml) was spread on Mueller-Hinton Agar (diameter, 90 mm) (Merck), by the disc diffusion method for the following antimicrobial agents (Mast/Rosco) with concentrations: Streptomycin (10 μg), Tobramycin (10 μg), Gentamicin (10 μg) Kanamycin (10 μg), Imipenem (10 μg) and Meropenem (10 μg) were then placed on the agar plate and incubated at 37°C for 24 h. The diameters of the zones of inhibition were measured and reported in mm.

Isolates of *A. baumannii* were defined as multidrug-resistant (MDR) when the organism was resistant to at least one agent in three or more antimicrobial categories that would otherwise serve as treatments for *Acinetobacter* infection.

An isolate was considered extensively drug-resistance (XDR) when it was non-susceptible to one or more of agent in all but 2 or less the categories. Pan drug resistant (PDR) was defined as non-susceptibility to all antimicrobial agents [17].

### DNA extraction, synthetic primers and PCR Assay

DNA extraction was carried out by commercial DNA extraction kit (CinnaGen, Iran).The presence of bla ^OXA-23^Carbapenemases gene was detected by PCR. PCR was performed in a standard enzyme Taq DNA polymerase. Single primer pair was used to amplify *bla*^*OXA-23*^ gene target fragment based on GenBank. The primers sequences as follows: Primer F: CCGTCGTTTACGACATTCA and bla^OXA-23^ R: AAAGAGCGCATTGCTTTGAT. For PCR reaction we used CinnaGen Master Mix and reactions were performed in a final volume of 25 μAccording to Primer (10 μmol, DNA template (50 ng). PCR was performed in a standard enzyme PCR system (Cinnagen) concentration reaction, Master Mix (1x) each, primer, template DNA 50 ng. The mixtures were incubated for 4 min at 94^oc^ for primary denaturation, 60 sec at 94^oc^ for secondary denaturation of the target DNA and then, annealing at 50^oc^ for 1 min, and extension at 72^oc^ for 1 min that 35 cycle was performed. The amplified products were analyzed by electrophoresis on 1% agarose gel (cinnagen) containing 0.1 g of ethidium bromide per ml in TAE buffer. The PCR product was visualized under UV light and photographed.

To determine the frequency of IS_*Aba*_ elements sets of primers (IS_*Aba*_1F/OXA-23R and IS_*Aba*4_F/OXA-23R) was used, respectively. PCR primers were designed using Primer 3 plus software (version 4.0; http://primer3.wi.mit.edu/; accessed 05.06.11) with sequences IS_*Aba*_ 1 F: TGAGATGTGTCATAGTATTC, IS_*Aba*_ /OXA^23^ R: AGAGCATTACCATATAGATT. PCR assays were performed under standard conditions.

## Results

In total, 100 *Acinetobacter baumannii* isolates were isolated from patients that were admitted to Intensive Care Unit and burned patients in Tehran and Sari‚ (Iran). During 2013, we observed that, 62% isolates showed resistance to aminoglycosides and carbapenems. Based on the susceptibility of the isolated *Acinetobacter baumannii* to number of antibiotics, was observed minimum resistance against Imipenem (67%) and Meropenem (74%) and maximum resistance against Streptomycin (90%), Gentamicin (83%), Tobramycin (83%) and kanamycin (80%).The results of PCR screening from 61 *Acinetobacter baumannii* resistant to aminoglycosides and carbapenems, 41(67%) isolates have bla ^OXA-23^ carbapenemase gene and IS_*Aba*_ (32.2%).

## Discussion

Nosocomial infections caused by multidrug resistant strains of *Acinetobacter baumannii* (MDR-AB) are currently among the most difficult to treat, and they continue to present serious challenges to clinicians’ empirical and therapeutic decisions in burned patient [1]. Outbreaks of extensively, and pan drug-resistant *A. baumannii* (XDR, and PDR, respectively) currently has been reported from worldwide. In this study, the high prevalence of XDR and PDR *A. baumannii* isolates (37.1% and 8.1%, respectively) from burned patient is consistent with previous reports.

The present study revealed that 17.8% vs 41.8% and 11.3% vs 0%of *A. baumannii* isolates from burned patients are resistant to Tigecycline and Colistin, respectively, by comparison with the other studies in Iran [1,18].

*Acinetobacter baumannii* is one of the most important nosocomial pathogens, able to cause severe infections, occurring principally in immunosuppressed patients, in patients with serious underlying diseases, or subjected to invasive procedures and treatment with broad-spectrum antibiotics. *A. baumannii* isolates resistant to various classes of antibiotics are emerging worldwide, and the recent resistance or reduced susceptibility to carbapenems, is considered aserious clinical problem due to the role of first choice therapy that these drugs have had until now [19]. *A. baumannii* isolates resistant to various classes of antibiotics are emerging worldwide, and the recent resistance or reduced susceptibility to carbapenems, is considered as serious clinical problem due to the role of first choice therapy that these drugs have had until now.Demonstrated that *A. baumannii* was the third pathogen for severity in high-risk wards, frequently derived from nosocomial pneumonia or bacteremia [10].

In a timely manner, antimicrobial resistance surveillance and strict infection control strategies are still lacking in burn ward in Iran, despite the alarming emergence of MDR-AB strains, particularly among those isolates that are not susceptible to Colistin. The emergence of Colistin-resistant *A. baumannii* in this study might be due to differences in *A. baumannii* strains available for study or increasing empirical use of Colistin. However, it is still unknown whether this obvious resistance to Colistin in *A. baumannii* isolates from burned patients in Iran was due to increase of *A. baumannii* virulence or other intervening factor. Interestingly, all Colistin resistant isolates were susceptible to Tigecycline and/or Tobramycin. This is very important for treating serious infections caused by Colistin resistance isolates. However, this combination still needs to be validated in animal model and clinical trials. The results of this study are consistent with a recent report in which a number of combinations exhibited potent activity against multidrug resistant strains of *A. baumannii* (MDR-AB) [20].

Although the presence of IS_*Aba*_ to play a substantial role in antimicrobial resistance in MDR-AB isolates, according to the best of our knowledge there is no data on the presence of IS_*Aba*_ in MDR-AB strains isolated from burn patients in Iran. However, further investigation is required to recover various IS_*Aba*_ in MDR-AB strains isolated from Iran.

This research showed that, it was notified that, the *Acinetobacter baumannii* resistant to aminoglycoside and Carbapenem in Tehran and Sari are rising dangerously. We have documented those almost more than half strains are resistant to carbapenems and aminoglycosides. Also we observed that resistance to aminoglycosides was higher than carbapenems.Carbapenem-resistance in these strains is due to the presence of bla ^OXA-23^ gene and we show that this gene, carried in 67%.

In conclusion, our data support that Tigecycline alone or plus Tobramycin exhibited a potent activity against Colistin-resistant *A. baumannii* isolates from burned patients. *Acinetobacter baumannii* strains isolated from Iranian burned patients are so heterogeneous and this is the first report of spreading of IS_*Aba*_ in the upstream of *bla*^OXA^ genes in MDR-AB isolates among burned patients in Iran. We also show that MDR-AB integrons were present among burned patients in Iran at almost the same time as they were described worldwide.

*Acinetobacter baumannii* strains isolated from Iranian burned patients are so heterogeneous and this is the first report of spreading of IS_*Aba*_ in the upstream of *bla*^OXA^ genes in MDR-AB isolates belonging to burned patients in Iran. Antimicrobial resistance surveillance and strict infection control strategies are still lacking in burn ward and ICU in Iran, despite the alarming emergence of MDR-AB strains, particularly among those isolates that are not susceptible to Colistin. The results of this study are consistent with a recent report in which a number of combinations exhibited potent activity against multidrug resistant strains of *A. baumannii* (MDR-AB).
